# Study protocol: a randomised controlled trial of a theory-based online intervention to improve sun safety among Australian adults

**DOI:** 10.1186/1471-2407-14-162

**Published:** 2014-03-07

**Authors:** Cathy M Cleary, Katherine M White, Ross McD Young, Anna L Hawkes, Stuart Leske, Louise C Starfelt, Kylie Wihardjo

**Affiliations:** 1 School of Psychology and Counselling, Queensland University of Technology, Victoria Park Road, Kelvin Grove, Brisbane 4059, Australia; 2 Faculty of Health, Queensland University of Technology, Victoria Park Road, Kelvin Grove, Brisbane 4059, Australia; 3 School of Public Health and Social Work, Queensland University of Technology, Victoria Park Road, Kelvin Grove, Brisbane 4059, Australia; 4 School of Public Health, Tropical Medicine, and Rehabilitation Sciences, James Cook University, Townsville 4811, Australia

**Keywords:** Sun protection, Theory of planned behaviour, Online intervention, Sun-protective behaviour, Adult, Oncology, Skin cancer

## Abstract

**Background:**

The effects of exposure to ultraviolet radiation are a significant concern in Australia which has one of the highest incidences of skin cancer in the world. Despite most skin cancers being preventable by encouraging consistent adoption of sun-protective behaviours, incidence rates are not decreasing. There is a dearth of research examining the factors involved in engaging in sun-protective behaviours. Further, online multi-behavioural theory-based interventions have yet to be explored fully as a medium for improving sun-protective behaviour in adults. This paper presents the study protocol of a randomised controlled trial of an online intervention based on the Theory of Planned Behaviour (TPB) that aims to improve sun safety among Australian adults.

**Methods/Design:**

Approximately 420 adults aged 18 and over and predominantly from Queensland, Australia, will be recruited and randomised to the intervention (n = 200), information only (n = 200) or the control group (n = 20). The intervention focuses on encouraging supportive attitudes and beliefs toward sun-protective behaviour, fostering perceptions of normative support for sun protection, and increasing perceptions of control/self-efficacy over sun protection. The intervention will be delivered online over a single session. Data will be collected immediately prior to the intervention (Time 1), immediately following the intervention (Time 1b), and one week (Time 2) and one month (Time 3) post-intervention. Primary outcomes are intentions to sun protect and sun-protective behaviour. Secondary outcomes are the participants’ attitudes toward sun protection, perceptions of normative support for sun protection (i.e. subjective norms, group norms, personal norms and image norms) and perceptions of control/self-efficacy toward sun protection.

**Discussion:**

The study will contribute to an understanding of the effectiveness of a TPB-based online intervention to improve Australian adults’ sun-protective behaviour.

**Trials registry:**

Australian and New Zealand Trials Registry number ACTRN12613000470796

## Background

Australians represent a high-risk group for the development of skin cancer, living in a country which has the joint highest incidence of skin cancer in the world [1], with two out of three Australians expected to develop skin cancer by the age of 70 years [2]. Melanoma and non-melanoma skin cancer combined account for approximately 80% of all new cancers diagnosed in Australia every year [3]. Specifically, incidence and mortality rates for melanoma in Australia are the highest in the world, with over 11,500 new cases diagnosed in Australia in 2009, including 3,000 people in the state of Queensland. Melanoma of the skin is the third most commonly diagnosed cancer in both Australian males and females (excluding non-melanoma skin cancer), with incidence rates continually increasing over the previous 3 decades [1]. This trend is illustrated by an increase of 42% in the melanoma incidence rate for males and an increase in the melanoma incidence rate of 18% for females between 1991 and 2009 [1]. Because of its high incidence, non-melanoma skin cancer (NMSC) also represents a significant burden on the Australian health budget. NMSC accounted for 950,000 general practitioner consultations in 2007 [4] and was listed as the most common reason for hospitalisation with the principal diagnosis of cancer in 2010-2011, with 95,312 people hospitalised [1].

Exposure of the skin to ultraviolet radiation [5,6] accounts for 95 to 99% of skin cancer diagnoses in Australia [3]. Most skin cancers are preventable by encouraging consistent use of sun protection methods including using a broad spectrum water resistant sun protection factor (SPF) 30+ sunscreen, staying in shady areas and limiting time in the sun between 10 am and 3 pm, and wearing a wide brimmed hat, sunglasses, and protective clothing to reduce sun exposure and sunburn [7].

Despite the potential of sun-protective behaviours to prevent skin cancer, the most recent data show that the majority of Australian adults are failing to adopt sun-protective behaviours [8-10]. The 2010-2011 National Sun Protection Survey found that only 19% of adults wore clothing with longer arm-cover during periods of peak sun exposure, 37% of adults used sunscreen, and 45% wore hats [11]. Wearing sunglasses was the most commonly adopted sun-protective behaviour among adults with 57% use. Exposure to the sun resulting in sunburn over the preceding weekend was reported by 13% of adults in this survey. A further study examining the incidence of sunburn among adults in the state of Queensland over the summer months found one in eight men and one in 12 women in Queensland reported being sunburnt on the previous weekend [12].

The human and economic burden of skin cancer in Australia provides an important impetus for research that informs health promotion interventions. Previous research and health change interventions in the field of adult sun protection has predominantly focused on measuring the adoption of sun-protective behaviour and raising awareness of the health implications of ultraviolet exposure and the means of reducing sun exposure [13]. While knowledge and awareness of risk have significantly increased over the last decade, recent findings suggest that these increases are not currently translating to adequate sun protection, a reduction in incidence of sunburn and skin cancer, or improved attitudes [13,14].

The socio-cognitive factors underpinning adult Australians’ decision-making about sun-safe practices have not yet been fully established [8] and the existing research falls short of providing a comprehensive model to address the complexity of behaviour change and to fully understand the motivations behind adults’ sun-protective decision-making. Understanding Australians’ sun-protective behaviour decision-making is critical to the development of theory-based interventions to increase sun-protective behaviour and effectively halt the trend in increasing incidence of skin cancer in Australia. The Theory of Planned Behaviour (TPB; [15]) offers a model of behaviour prediction useful not only in understanding sun protection decision-making but also in informing intervention development.

### Theoretical framework

**Figure 1:**
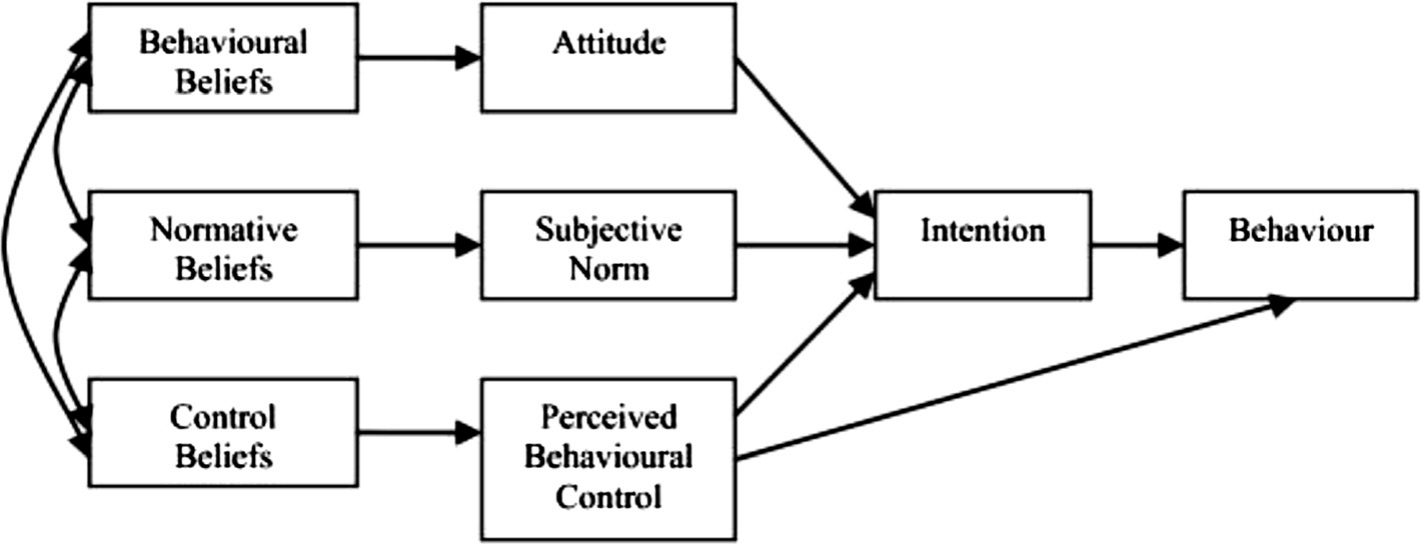
**The theory of planned behaviour (TPB) **[15]**.**

The TPB [15] is a well-validated decision-making model that has been used to successfully understand a range of social and health-related behaviours [16-22]. Specifically, the effectiveness of the model’s application to predicting and understanding sun-protective behaviour has been demonstrated in Australia [18,20,23,24] and internationally [17,19]. In the model (see Figure 1), behavioural intention is the most proximal determinant of the target behaviour. Attitudes (positive and negative behavioural evaluations), subjective norms (perceived pressure from important referents to perform the behaviour), and perceived behavioural control (PBC; perceptions of control over performing the behaviour/perceived ease or difficulty in performing the behaviour), in turn, exert an impact on behaviour via behavioural intention. PBC is also conceptualised as a direct predictor of behaviour [15]. The underlying cognitive belief-base of attitudes, subjective norms, and PBC are behavioural (costs and benefits), normative (specific referents’ approval or disapproval), and control (barriers and facilitators) beliefs, respectively. The relative strength of the predictors in the model are expected to vary depending on the behaviour under study; based on 185 applications of the TPB across a range of behaviours [25], attitudes, subjective norms, and PBC together explained an average of 39% of the variance in intention, with intention accounting for an average of 27% of the variance in behaviour (and a further 2% of variance attributable to PBC).

Ajzen [15] describes the TPB as a model open to the inclusion of additional predictors provided that there is strong theoretical justification for their inclusion and that the predictors explain an adequate amount of unique variance. Accordingly, extensions to the TPB have been proposed to make the model applicable in a range of different contexts but, also, to address conceptual and measurement issues with the relatively weak normative construct. Subjective norm is repeatedly found to be the weakest predictor of intention [25], which has led some researchers to propose a re-conceptualisation of this construct or extensions to the TPB to incorporate other normative influences. In the sun safety literature, researchers have suggested broadening the normative component of the model with the addition of group norms [23,26], image norms [16], and personal norms [27]. Informed by a social identity [28] and self-categorisation approach [29], group norms aim to capture the perceived expectations and actions of members of specific, salient, in-groups. The in-group that is salient for a particular behaviour is situation-specific and will, as such, vary across contexts. Group norma reflect a *prescriptive* rather than a *descriptive* normative influence and comprise two components: behavioural norms, which are the perception of whether group members perform the behaviour, and group attitudes, which are the perception of group members’ evaluation of the behaviour. In the TPB, a behaviour that is typically performed and highly valued by members of a salient in-group is, thus, thought to strengthen behavioural intentions. Extended TPB models that have incorporated group norms have received recurring support in the literature (e.g., [23,30,31]). In the context of sun safety, White et al. [20] found that the perceived group norms of friends had a direct influence on young Australians’ sun-protective intentions and behaviour.

Image norms are another normative influence potentially relevant to people’s sun-protective intentions and behaviour [16]. These norms are the cognitive representations of stereotypical members of particular groups (e.g., tanned and non-tanned people), and reflect individuals’ self-presentational concerns about their image [16]. For instance, perceptions that a tan is attractive and healthy might lead individuals to deliberately expose themselves to the sun without using sun protection to develop a tan. Image norms are thought to represent the values of society in general (e.g., as portrayed in the media). Previous attempts to modify image norms have focused on altering normative perceptions about the attractiveness of being tanned [16]. Jackson and Aiken [32] also suggest that increasing the perceived attractiveness of pale image norms may assist in improving sun-protective behaviours.

The concept of personal norms has also been proposed as an addition to the normative component of the TPB (e.g., [15]). Personal norms are regarded as an individual’s own values as they relate to performing a certain behaviour [33]. While the performance of some behaviours may be linked to moral or ethical values (i.e., moral norms), self-identity can also influence the formation of personal norms. For instance, while individuals may not feel any moral obligation to perform sun-protective behaviours, they may regard themselves as a responsible person and, therefore, engage in behaviours which are perceived to reduce risk (i.e., sun safety, avoidance of sunburn). Personal norms differ from self-identity, however, in that it originates more from personal rather than societal values [33].

To target influential determinants of sun protection intentions and behaviour, this online intervention builds on two previous studies undertaken by the authors. A qualitative elicitation study (N = 42) (Leske S, Young RM, White KM, Hawkes AL: A qualitative exploration of sun safety beliefs among Australian adults, forthcoming) was conducted to identify relevant costs and benefits of sun protection, important referent groups, and barriers and facilitators to sun protection. The findings of the qualitative study were then used to develop measures for a large-scale prospective study (N = 579) to assess the relative predictive utility of the TPB predictors and additional social, personal, and normative influences on Australian adults’ sun-protective behaviour (White KM, Starfelt LC, Young RM, Hawkes AL, Leske S, Hamilton K: Predicting Australian adults’ sun-safe behaviour: Examining the role of personal and social norms, submitted).

Critical beliefs influencing sun protection identified by the authors in previous research informed the development of the current intervention. Hamilton et al. [24] found people were more likely to sun protect if they believed long-sleeved shirts and hats were fashionable, were influenced by friends’ favourable attitudes towards sun protection, and believed they were less likely to tan if practising sun protection. Further, predictors of non-adoption of sun-protective behaviours which will be incorporated into this study are the perception that sun protection was inconvenient and easy to forget. Additional influences identified based on qualitative data have been incorporated into the intervention, namely the role of personal choice/responsibility in the decision to engage in sun-protective behaviour and the belief that being in the sun and having a tan are part of Australian identity and culture.

Computer-based interventions have been used to target behaviour change in a wide range of health issues over the last decade and provide a means of administering economical and easily accessible interactive health interventions which are far reaching within the population [13,34]. Research by Cugelman et al. [34] found that, compared with waitlists, online interventions have demonstrated moderate efficacy while, compared with print materials, they offer similar impacts but with the advantages of lower costs and broader reach. Further, research by Webb et al. [35] found that more extensive use of theory, and specifically online interventions based on the TPB, tended to have more substantial effects on behaviour. Despite their demonstrated efficacy in producing health behaviour change, online, multi-behavioural, theory-based interventions have yet to be explored fully as a medium to target adults’ sun-protective attitudes, beliefs, and sun-protective behaviour within the Australian context.

Limited research has examined the efficacy of online/web-based interventions in increasing a specific sun-protective behaviour (e.g., sunscreen use; [36]); however, there is a particular dearth of theory-based, online interventions targeting multiple sun-protective behaviours. We hypothesise that adults exposed to the online intervention will report an increase in positive sun-protective attitudes, normative support, and self-perceptions of control/self-efficacy, leading to increased sun protection intentions and behaviour, compared with participants in both an information only and control group (measurement only).

This paper presents the study protocol for an online intervention aimed at improving sun-protective behaviour in adults. The research will use an extended version of the TPB to develop and test the efficacy of an online sun-protective intervention derived from this approach. The intervention will target previously identified attitudes toward sun protection, normative influences, and barriers and motivators, as well as targeted aspects of personal choice/responsibility, and tanning being part of Australian identity.

## Methods/Design

### Study design

The study is a three-armed prospective randomised controlled trial targeting approximately 420 males and females aged 18 years or older and living predominantly in the state of Queensland. An online intervention was considered to be potentially useful in this geographical area given that Queensland is a state where access to services is limited in regional and rural areas. Consenting participants will be randomised in a 200:200:20 ratio to (a) the intervention or (b) information only or (c) a control group using a computer-generated random number sequence. Randomisation will be undertaken by the consultant project web developers in association with the project investigator. Participants in each of the groups will complete three online assessments; at baseline, one week, and one month after the initial survey. Participants randomised to the intervention and information only groups will complete a brief survey immediately following completion of their respective conditions to measure each of the main study constructs.

### Study aim

The aim of this study is to evaluate the effectiveness of a TPB-based online sun safety intervention in increasing positive attitudes, normative support, and perceptions of self-efficacy/control, leading to increased sun protection intentions and behaviour in adults.

### Study sample

#### Sample eligibility criteria and recruitment procedures

Eligibility criteria will include male and female adults (aged 18 or over) living in Australia. Participants will be recruited from the community through university-based media releases, community billboards, newsletters, email lists, snowball sampling techniques, and the use of an existing database of participants from a previous sun safety study who consented to be contacted for participation in future studies.

Participants will receive an email and flyer providing information about the study and a link to the study website. Consent to participate will be obtained after participants are presented with a comprehensive outline of the study online and will involve participants clicking a box indicating that they agree to participate in the study. Participants will be randomised to a study condition immediately after completing Questionnaire 1. A link to the post-intervention questionnaires will be emailed to participants a week and then one month after the initial questionnaire.

Participants are advised that they will be eligible to receive an AUD $20 store voucher after completion of Questionnaire 1 and another AUD $20 store voucher after completing the two follow up questionnaires 1 week and 1 month later.

#### Sample size

It is aimed to recruit a total of 420 participants (200 intervention/200 information only/20 control). Based on our previous research in the area, it is anticipated that there will be approximately 35% attrition over 4 weeks of follow-up for reasons such as failure to complete follow-up questionnaires. A total sample of approximately 260 (420–140) completing participants (130/group) is required to detect a medium effect in sun-protective behaviour. This sample size was determined by power analysis using the G*Power program [37,38]. Significance level (alpha) was established at 0.05 to avoid a Type 1 error, power (1–beta) was set at 95% to avoid a Type II error, and effect size was determined at .25. Therefore, for a 95% chance of detecting as significant a 4 week difference in sun safe behaviour, approximately 130 participants in each group are needed to complete the study.

### Study conditions

#### Intervention

The intervention is computer-based and will be conducted in the participants’ homes or in their chosen location based on accessibility to the online intervention. The single session interactive intervention will take approximately 20-25 minutes to complete and will address three main constructs related to sun protection.

The first construct, sun protection-related attitudes and beliefs, will be targeted through a series of questions and quizzes in which participants will be asked to consider advantages and disadvantages of sun protection as well as common misconceptions about sun protection. The second construct, fostering perceptions of friendship group normative support for sun protection, will be addressed through the use of animated scenarios depicting situations in which a character is faced with opposition to performing sun-protective behaviour from an important referent or referents. A series of questions will prompt participants to consider how they would respond in each situation and how they could prevent the situation from occurring. An increase in perceptions of control/self-efficacy with using sun protection is the third construct addressed in the intervention and is addressed by a set of animated scenarios and accompanying questions which ask participants to consider specific barriers to sun-protective behaviour and to suggest solutions to these barriers. Additionally, participants will be prompted to set a specific sun safe goal, to identify barriers to success, and to propose solutions to the barriers. Participants will be asked to create a contract online which outlines their intentions to overcome these barriers and will be provided with an option to print/save or email the contract to a friend. Further to these constructs, participants will be prompted in the intervention to consider their attitudes to tanning (including culturally-based as an Australian) and issues related to personal responsibility to engage in sun protection.

#### Information only

The information only group will be conducted at participants’ homes or preferred location with access to a computer. Participants will be asked to view an 8 minute online DVD and three fact sheets relating to sun-protective behaviour which are currently available from Cancer Council Queensland’s website. The DVD is aimed at providing practical advice to adults to reduce their risk of developing skin cancer through prevention and early detection. Topics include skin cancer, types of skin cancers, means of protecting against sun exposure, UV index, and early detection including self-examination. The fact sheets cover the topics of skin cancer, sunscreen, and myths about sun protection. Participants will be asked to confirm that they had read all three fact sheets.

#### Control

Control participants will not be required to do anything beyond completing the three online surveys.

### Study and data integrity

The study design will be guided by the CONSORT (Consolidated Standards of Reporting Trials) statement [39].

### Measures

Data will be collected by self-reported pre- and post-intervention questionnaires developed by the researchers and using standard TPB items. The pre-intervention questionnaire will take approximately 15-20 minutes to complete and will be completed online immediately before the online intervention or information only session.

The post-intervention questionnaires will be completed online immediately following the intervention and at one week and four weeks after the intervention. The post-intervention questionnaires will assess the same constructs as Questionnaire 1, plus an additional set of questions which measure exposure to other sun-protective behaviour materials or promotions in the preceding week (Questionnaire 2) and month (Questionnaire 3).

#### Variables

Demographic data collected pre-intervention will include age (in years), sex (male or female), and postcode. Data will also be collected on colour of skin before tanning (pale white skin, white skin, light brown skin, moderate brown skin, deep dark brown to black skin), colour of skin with repeated exposure to the sun without protection (get no sun tan at all or occasionally get freckled, get mildly or occasionally tanned, get moderately tanned, go very brown and deeply tanned), natural hair colour (black, dark brown, light brown, dark blonde, light blonde, red), eye colour (dark brown, light brown, green, blue), number of hours per week of work conducted outdoors, and hours spent in the sun in the past week. Data relating to level of confidence using computers and frequency of accessing health information on the internet will also be gathered.

#### Outcome measures

Primary outcomes variables will assess the effectiveness of the online intervention in improving participants’ self-reported sun-protective intentions and behaviour.

The target behaviour is “performing sun-protective behaviours (i.e., using SPF 30 + sunscreen, wearing protective clothing such as a hat, long-sleeved shirt and sunglasses, and seeking shade between 10 am and 3 pm) every time you go in the sun for more than 10 minutes during the next week” (Table 1).

**Table 1:** Primary and secondary outcome measures

**Variable**	**Number of items**	**Scale**	**Measurement strategies**
**Primary outcome variables**			
Intentions	3	1 (strongly disagree) to 7 (strongly agree)	“I intend to perform sun-protective behaviours.”; “I plan to perform sun-protective behaviours.”; “It is likely that I will perform sun-protective behaviours.”
Behaviour	3	1 (never) to 7 (always)	“Think about the past week. In general, how often did you perform sun-protective behaviour?”; “Think about the past week. On average, how often did you perform sun-protective behaviours on Saturday and Sunday?”; “Think about the past week. On average, how often did you perform sun-protective behaviours on a typical week day?”
**Secondary outcome variables**			
Attitudes	6	1 (pleasant) to 7 (unpleasant)	“Performing sun-protective behaviours every time I go in the sun for more than 10 minutes during the next week, would be…” (reverse scored)
		1 (good) to 7 (bad)	
		1 (wise) to 7 (unwise)	
		1 (easy) to 7 (difficult)	
		1 (nice) to 7 (awful)	
		1 (positive) to 7 (negative)	
Subjective Norms	3	1 (strongly disagree) to 7 (strongly agree)	“Those people who are important to me would want me to perform sun-protective behaviours.”; “Most people who are important to me would approve of me performing sun-protective behaviours.”; “Most people who are important to me would think that I should perform sun safe behaviours.”
Perceived Behavioural Control	4	1 (strongly disagree) to 7 (strongly agree)	“I have complete control over whether I perform sun-protective behaviours.”; “It is mostly up to me whether I perform sun-protective behaviours.”; “If I wanted to it would be easy for me to perform sun-protective behaviours.”; “I am confident that I could perform sun-protective behaviours.”
Group Norms	4	1 (strongly disagree) to 7 (strongly agree)	“Most of my friends perform sun-protective behaviours.”; “My friends think that performing sun-protective behaviours is a good thing to do.”; “How many of your friends would think that performing sun-protective behaviours every time you are out in the sun for more than 10 minutes in the next week is a good thing to do?”; “How many of your friends would perform sun-protective behaviours every time they are out in the sun for more than 10 minutes during the next week?”
		1 (none) to 7 (all)	
Personal Norms	2	1 (strongly disagree) to 7 (strongly agree)	“I think I should perform sun safe behaviours.”; “Performing sun safe behaviours is something I should do.”
Image Norms	5	1 (strongly disagree) to 7 (strongly agree)	“Celebrities and movie stars always seem to have a tan.”; “I see more examples of models who do not have a tan on TV and in magazines than I used to.” (reverse scored); “I think that to be a successful movie star or TV star you should have a tan.”; “It seems that society wants people to have a tan.”; “I can think of many movie stars and TV stars who do not have a tan” (reverse scored).
Tanning	2	1 (strongly disagree) to 7 (strongly agree)	“A person with a tan looks Australian”; “A person without a tan looks ‘Un-Australian”.
Responsibility	3	1 (strongly disagree) to 7 (strongly agree)	“I think it is my responsibility to perform sun safe behaviours”; “I think it is up to the government to ensure that sun safety measures are available” (reverse scored); “It is my personal choice to perform sun safe behaviours”.

Secondary outcome variables will assess the intervention’s effectiveness in improving participants’ attitudes toward sun protection; participants’ perceptions of normative support for sun protection (i.e. subjective norms, group norms, personal norms and image norms); and participants’ perceptions of control/self-efficacy toward sun protection (PBC). Additional constructs identified in previous research will also be examined, namely participants’ perceptions, as an Australian, of tanning and their perceptions of personal responsibility to engage in sun protection.

### Ethical considerations

The protocol of this paper was approved by the Queensland University of Technology Human Research Ethics Committee (approval number: 1200000658).

### Data analyses

Chi-square (categorical variables), ANOVA (normally distributed continuous variables), and Kruskal-Wallis tests (non-parametric variables) will be used to compare baseline characteristics between groups, as well as between those with complete data and those who withdrew or were lost to follow-up. Outcomes will be analysed using general linear models for each of the change outcomes, including the main effects of group and time and the interaction of group and time. Sensitivity analyses will be conducted to determine the effect of missing data.

## Discussion

This study investigates the efficacy of a TPB-based multi-behavioural online intervention to promote adults’ sun-protective behaviour. The intervention, which incorporates previously identified psycho-social factors relevant to Australian adults’ sun safe decisions, will examine the efficacy of addressing people’s attitudinal beliefs about sun protection and tanning, considering the social approval of important referents, and tackling the barriers to sun protection in promoting more regular performance of sun safety measures and, consequently, combating the current rates of skin cancer for Australian adults. The strengths of this trial include its use of an established theoretical model to both inform and evaluate a health intervention which targets each of the behaviours integral to sun protection. Theory-based interventions which are effective in promoting sun-protective behaviours are critical to combating the increasing rates of skin cancer. This evidenced-based online intervention could provide an economical, easily accessible, far reaching means of targeting current lack of engagement in sun-protective practices and reducing sun exposure within a high-risk population. If effective, the intervention will contribute to increased sun-protective behaviour that is critical for reducing the incidence of skin cancer. At an individual level, this could equate to improving quality of lives while, at a national level, it could contribute to reducing the economic burden of skin cancer and improve longevity.

## Abbreviations

SPF: Sun protection factor; TPB: Theory of planned behaviour; UV: Ultraviolet; ANOVA: Analysis of variance.

## Competing interests

The authors declare that they have no competing interests.

## Authors’ contributions

KMW, RY, and AH conceptualised the study. CC, KMW, RY, AH, and SL further developed the study protocol and are responsible for the implementation of the intervention. CC, with assistance from SL and LS, was responsible for drafting the manuscript and all authors contributed to the revision of the manuscript and accept responsibility for and approve of the final version.

## Pre-publication history

The pre-publication history for this paper can be accessed here:

http://www.biomedcentral.com/1471-2407/14/162/prepub
